# Social Integration and Depression of Older Adults in Rural China: Do Family or Friendship Ties Make a Difference?

**DOI:** 10.1093/geroni/igaa057.1932

**Published:** 2020-12-16

**Authors:** Dan Zhang, Zhiyong Lin, Shuzhuo Li

**Affiliations:** 1 Xi’an Jiaotong University, college park, Maryland, United States; 2 University of Texas, Austin, Austin, Texas, United States; 3 Xi’an Jiaotong University, Xi’an, Shaanxi, China

## Abstract

Despite increasing acknowledgement that social integration/isolation is an important determinant of health in later life, relevant evidence for older adults in less developed social contexts is still limited. Data derived from 2015 and 2018 waves of a longitudinal study of 976 older adults, aged 60 and older, living in rural areas of Anhui Province, China. We analyzed how the level of social integration/isolation (measured as family and friendship ties) impacted depressive symptoms of older adults. Our results showed that more than half of older adults in our sample were either isolated from family or friends. Further analysis demonstrated that older people who were isolated from friends were more depressed in comparison with those who were closely integrated into friendship ties, while no such association was found in relation to family ties. Assessments of social integration among older adults should account for both family and friendship ties.

